# Rotamers in Crystal Structures of Xylitol, D-Arabitol and L-Arabitol

**DOI:** 10.3390/ijms23073875

**Published:** 2022-03-31

**Authors:** Monika Wanat, Maura Malinska, Malgorzata Kucia, Rafal R. Sicinski, Krzysztof Woźniak

**Affiliations:** 1 Biological and Chemical Research Centre, Department of Chemistry, University of Warsaw, 101 Żwirki i Wigury, 02-089 Warszawa, Poland; mwanat@uw.edu.pl (M.W.); mmalinska@chem.uw.edu.pl (M.M.); m.kucia@student.uw.edu.pl (M.K.); 2 College of Inter-Faculty Individual Studies in Mathematics and Natural Sciences (MISMaP), University of Warsaw, 2C Stefana Banacha, 02-097 Warszawa, Poland; 3 Department of Chemistry, University of Warsaw, Pasteura 1, 02-089 Warszawa, Poland; rasici@chem.uw.edu.pl

**Keywords:** crystal structure, xylitol, rotamer, stereochemistry

## Abstract

Rotamers are stereoisomers produced by rotation (twisting) about σ bonds and are often rapidly interconverting at room temperature. Xylitol—massively produced sweetener—(2*R*,3*r*,4*S*)-pentane-1,2,3,4,5-pentol) forms rotamers from the linear conformer by rotation of a xylitol fragment around the C2–C3 bond (rotamer 1) or the C3–C4 bond (rotamer 2). The rotamers form two distinguishable structures. Small differences in geometry of rotamers of the main carbon chain were confirmed by theoretical calculations; however, they were beyond the capabilities of the X-ray powder diffraction technique due to the almost identical unit cell parameters. In the case of rotamers of similar compounds, the rotations occurred mostly within hydroxyl groups likewise rotations in L-arabitol and D-arabitol, which are discussed in this work. Our results, supported by theoretical calculations, showed that energetic differences are slightly higher for rotamers with rotations within hydroxyl groups instead of a carbon chain.

## 1. Introduction

Xylitol is a very important compound as it is used as a food additive and sugar substitute. Its European Union code number is E967. It naturally occurs in small amounts in plums, strawberries, cauliflower, and pumpkin; humans and animals make trace amounts during the metabolism of carbohydrates. Xylitol is widely used in sugar-free chewing gums, though it is mostly combined with sorbitol to lower the production costs. The global market for xylitol is estimated at 250 thousand metric tons, valued at >US$ 1 billion by 2022. Moreover, xylitol is often used in crystallographic studies as a model compound. For instance, crystal structures of xylitol were used for validation of quantum crystallography methods such as Hirshfeld Atom Refinement or Normal Mode Refinement [1,2,3,4,5]. Charge density studies on xylitol confirmed that thermal motions of atoms may be deconvoluted from the static electron density. [6] These studies also allow for a demonstration that entropy information may be obtained in crystallographic experiments [6]. Neutron studies of xylitol show that when a reliable rigid-body model is available, then the estimation of the H-atoms mean-square displacement parameters can be achieved [7]. Moreover, xylitol [8] and its eutectic mixtures (xylitol-erythritol and xylitol-arabitol) [9] can be used as heat-storage in heat devices as a melt or super-saturated solution. Derivatives of xylitol were also obtained in the studies on new surfactants for biomedical applications [10,11].

Rotational isomer, or rotamer, is a form of stereoisomerism in which the isomers can be interconverted just by rotations about formally single bonds. Rotation about the C2–C3 σ bond in butane results in conformation *anti* and *syn*. Two rotamers of cyclohexane are chair and boat conformations. These two forms can be interconverted by twisting the ring structure. Another example is amino-acid rotamers defined by sidechain torsion (Chi) that have a different probability of finding in protein structures. The rotation barrier depends on the electrostatic repulsion and steric hindrance between moving groups, e.g., in butane is below 20 kJ/mol.

**Figure 1:**
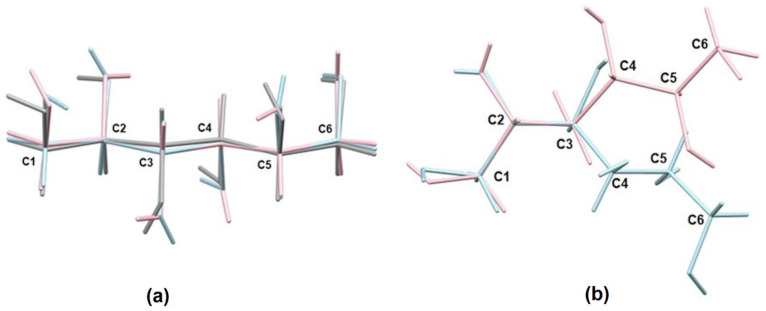
Overlay of rotamers of (**a**) D-mannitol—α (blue), β (pink), and δ (grey) [12]; and (**b**) sorbitol [13].

An example of rotamers of sugars in the crystal structures is polymorphic forms of D-mannitol i.e., α, β and δ [12]. The α and β forms crystallize in P2_1_2_1_2_1_ space group, whereas the δ form crystallizes in P2_1_ space group. The rotations for each molecule mostly affect orientation of hydroxyl groups that result in different hydrogen bond networks and, in consequence, different packing schemes (Figure 1a). The rotation of the carbon atom chain was found in the molecules in the asymmetric unit of the sorbitol monoclinic crystal structure, which is sickle-shaped and linear [13] (Figure 1b). In other polymorphs of sorbitol, only one form (non-linear) was crystallized in the orthorhombic space group—P2_1_2_1_2_1_ or P2_1_2_1_2 [14,15,16]. Moreover, in one of the non-linear forms that crystallized in the orthorhombic space group, rotations affected orientation of hydroxyl groups [16], and this is yet another rotamer.

**Figure 2:**
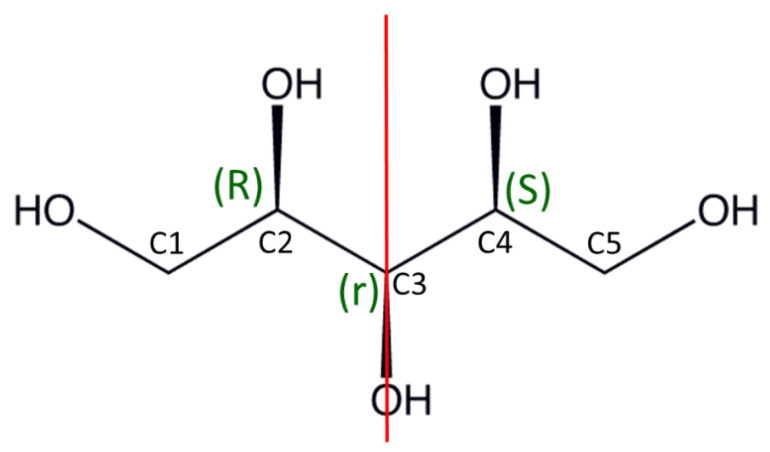
The structure of the xylitol molecule, atom numbering system and absolute configurations. The red line is the mirror plane.

**Figure 3:**
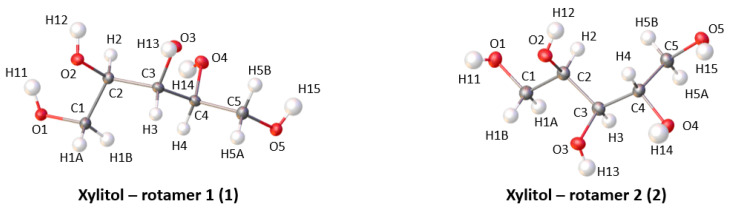
An ORTEP view of rotamers **1** and **2** of xylitol. Displacement ellipsoids are drawn at 50% probability level.

Xylitol molecule, (2*R*,3*r*,4*S*)-pentane-1,2,3,4,5-pentol as a linear conformer, has the *meso* configuration due to the presence of the mirror plane of symmetry (Figure 2). However, xylitol crystallizes in the non-centrosymmetric P2_1_2_1_2_1_ space group, and the xylitol molecule adopts a non-linear sickle-like conformation of the carbon chain [17]. Low-density monoclinic polymorph was reported [18]; however, the crystal structure remains unknown. The presence of different conformations in solution (D_2_O and (^2^H_5_)pyridine at 298K) of xylitol has been previously [19] suggested by application of the Karplus equation based on NMR spectroscopy studies of ribose [20]. This method enabled calculation of the vicinal proton–proton coupling constants from the corresponding dihedral angles. It was believed that in solution, the *trans* and *gauche* conformers of xylitol rapidly interconvert. Such free rotations of the xylitol molecule then led to a distribution of solution conformations that were not related to the crystal structure. Moreover, the results of ^13^C NMR spectroscopy measurements indicated that two (or more) equivalent non-linear rotamers could exist in the rapidly formed equilibrium [19]. For instance, xylitol in linear conformation was crystallized with xylose isomerase [21] and in sickle-like conformation with hyaluronate lyase [22]. Surprisingly, most deposited crystal structures of pure xylitol contain rotamer 2 (Figure 3) [1,5,6,7,17], whereas rotamer **1** was reported recently [23] with an unreliable Flack parameter. Nonetheless, in all deposited rotamer **2** crystal structures [5,6,7,17,24], planar zigzag C atom chain conformation for C1–C2–C3–C4 atoms was detected. This conformation leads to the occurrence of two hydrogen bond motifs: four-link infinite spirals (…O2–H2…O4–H4…) and six-link infinite spirals (…O5–H5…O1–H1…O3–H3…) [17].

In this paper, we describe crystal structures of xylitol with a planar C2C3C4C5 zigzag chain conformation for both rotamers confirmed by the use of the anomalous dispersion correction with copper X-ray diffraction experiment. The second rotamer of xylitol that we report here may have important biochemical and economic consequences as each year its production is worth more than 1 billion USD. Our study is supported by the analysis of crystal structures of L-arabitol and D-arabitol as other examples of rotamers. According to the literature, till now, X-ray studies have been performed for L-arabitol using X-ray powder diffraction technique [25]. With the aid of differential scanning calorimetry, the experiments enabled characterization of polymorphic forms of L-arabitol [26]. D-arabitol was characterized as well; however, ADPs, as well as Flack parameter, were not determined, and the absolute configuration in the cif file was determined as “unknown” [27]. Differences and similarities between the conformations of rotamers, supported by the theoretical calculations, will be discussed.

## 2. Results and Discussion

### 2.1. Crystal Structures of Xylitol

First, we clarify the numbering system used here to compare the different xylitol structures. According to the IUPAC nomenclature, the xylitol name is (2*R*,3*r*,4*S*)-pentane-1,2,3,4,5-pentol, i.e., the absolute configurations of C2 and C4 should be *R* and *S*, respectively. The configurations of these atoms using the atom-numbering scheme originally applied by authors of publications describing the structures **B**–**E** is *S* and *R*, respectively. The resulting name (2*S*,3*r*,4*R*)-pentane-1,2,3,4,5-pentol of the xylitol molecule is in conflict with commonly accepted rules of sugar nomenclature. It shows that carbon atoms in the deposited structures **B**–**E** are numbered in the reverse order, i.e., C1, C2, C4, and C5 should be named C5, C4, C2, and C1, respectively. For the comparative purposes, structures **B**–**E** presented here were renumbered to be consistent with IUPAC nomenclature.

**Figure 4:**
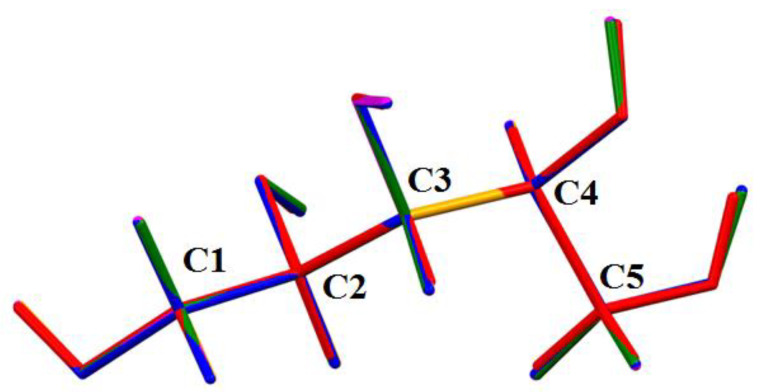
Overlay of all xylitol structures deposited in CSD. Xylitol structures were marked as red (XYLTOL), blue (XYLTOL01), orange (XYLTOL02), magenta (XYLTOL03), and green (XYLTOL04).

Crystal structures of xylitol deposited in CSD have the same absolute configuration despite the fact that the information about the Flack parameter was only available in one case (**D**). It appeared that known structures of xylitol were equivalent to **D** (Figure 4).

**Figure 5:**
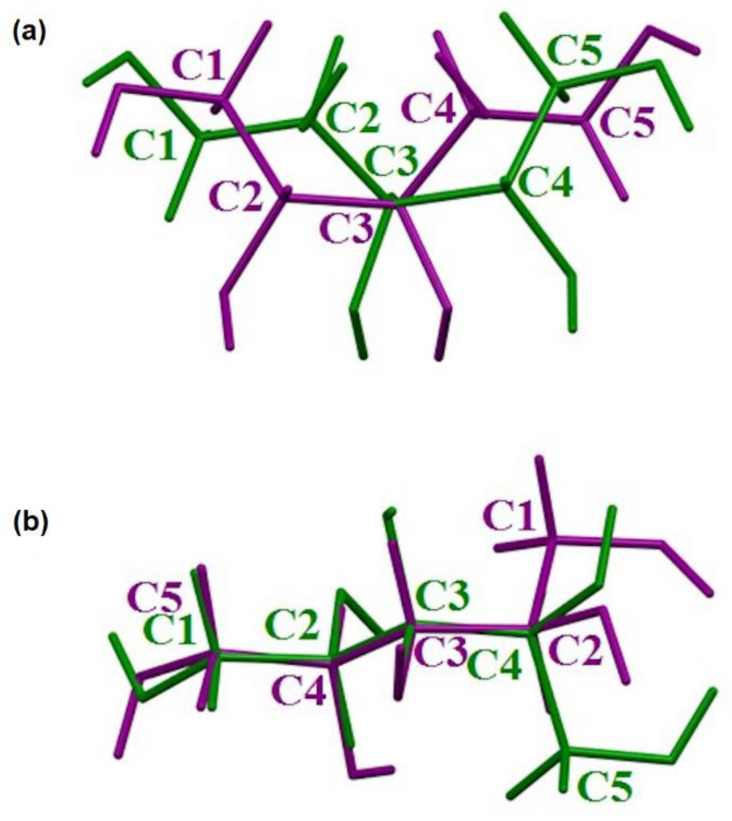
Overlay of both rotamers: (**a**) along C1–C2–C3–C4–C5 chains and (**b**) along C1–C2–C3–C4–C5 chain of one rotamer and reverse chain of second rotamer. Rotamers **1** and **2** are drawn as green and purple sticks, respectively.

**Figure 6:**
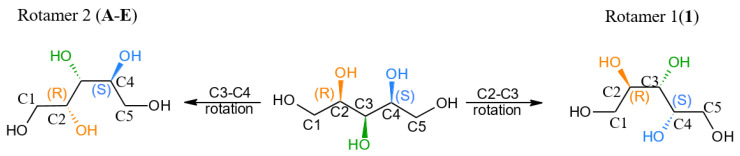
Molecular structures of the two rotamers of xylitol. To the best of our knowledge, Rotamer **1** is described for first time in this work (**1**). Rotamer **2** has been previously described (compounds **A**–**E**).

Here, we report the first crystal structure of another rotamer of xylitol (**1**) with an assigned absolute configuration using the anomalous signal. Surprisingly, comparison of **1** and **D** revealed that these structures could not be overlaid (Figure 5). The measurement of the xylitol single crystals provided by authors of the **D** structure [5] was repeated by us using copper radiation (structure **2**), which confirmed that indeed two different crystal forms of xylitol do exist. We confirmed that the result as the Flack parameters after refinements of structure **1** with the hkl dataset of **2** and vice versa were equal to 0.97(8) and 0.99(4), respectively. The xylitol molecule in **1** adopts a crystal planar zigzag chain C2, C3, C4, and C5 instead of C1, C2, C3, and C4. Apparently, both structures resemble a pair of enantiomers (Figure 6); however, it appears that this moiety is a *meso* compound, and there is no doubt that enantiomers of xylitol cannot exist. Careful analysis showed that starting from the linear form of xylitol, rotation around the C3–C4 bond resulted in the structure **D**, whereas a certain rotation around the C2–C3 bond yielded structure **1** (Figure 6). As a result, we can observe two structures with different xylitol rotamers. Moreover, the absolute configuration of the C3 atom is *r* according to the Cahn–Ingold–Prelog (CIP) priority rules because the *R* carbon atom takes priority over *S*.

Our further aim of this crystal analysis was to establish the contribution of crystals of particular rotamers in the crystalline sample. To achieve this, the X-ray powder diffraction data collection for xylitol was performed and results were normalized (Figure S1). Using Mercury, the powder patterns for both rotamers were calculated and normalized (Figure S2). As the differences between the unit cell parameters of two rotamers (Figure S3) were very small, the patterns for both rotamers with the same cell parameters were calculated. The patterns of the rotamers were used to analyze the differences between them. As a result, the slight differences can be observed in the intensity of peaks at a level of 3% (Figure S3). Due to the high similarity of the patterns of the rotamers, the final contribution of the crystals of the particular rotamers in the sample could not be estimated. Powder X-ray diffraction is considered to be a fingerprint-type technique; however, information about potential rotations of the compounds in sample could not be established with powder X-ray diffraction method.

### 2.2. Geometry

Rotamers **1** and **2** adopted different zigzag carbon-chain conformations. Comparison of the corresponding dihedral angles for both rotamers highlighted the many differences between the corresponding structural parameters (Table S1). Analysis of the C2→C3 and C3→C4 Newman projections (Figure S4) showed that due to the rotation around the C2–C3 and C4–C3 bonds, the six dihedral angles, which are in the *gauche* orientation in rotamer **1** (H2–C2–C3–O3, O2–C2–C3–H3, C1–C2–C3–C4, C2–C3–C4–O4, O3–C3–C4–C5, H3–C3–C4–H4), are in the antiperiplanar conformation in rotamer **2**. At the same time, the following torsion angles O2–C2–C3–C4, C1–C2–C3–O3, H2–C2–C3–H3, O3–C3–C4–H4, H3–C3–C4–O4, C2–C3–C4–C5 are in the antiperiplanar conformation in rotamer **1** and in the gauche conformation in rotamer **2** (Figure S4). More detailed description of geometry is available in the Supporting Information (Figure S4, Tables S1 and S2).

### 2.3. Crystal Structures of L-Arabitol and D-Arabitol

**Figure 7:**
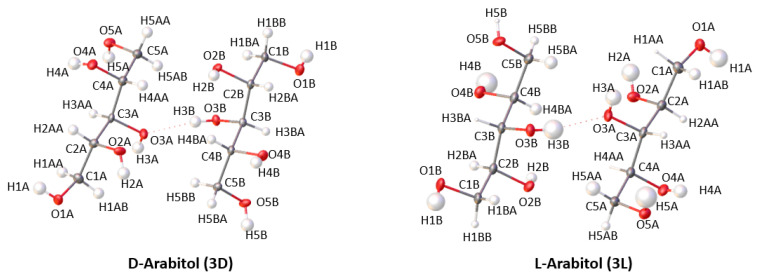
An ORTEP view of D-arabitol (**3D**) and L-arabitol (**3L**). Displacement ellipsoids are drawn at 50% probability level.

**Figure 8:**
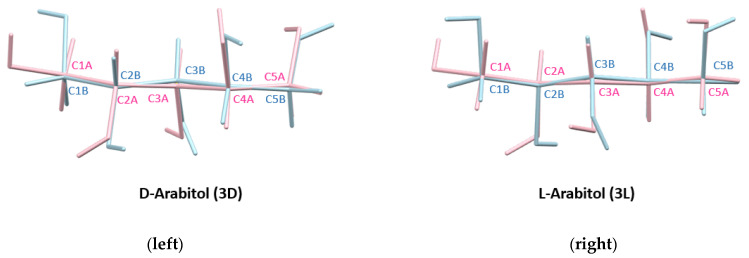
Overlay of molecules in the asymmetric unit cell of **3D** (**left**) and **3L** (**right**). For both crystal structures, **3L** and **3D**, the molecules in the asymmetric unit are rotamers.

Analysis of the crystal structures of L-arabitol (**3L**) and D-arabitol (**3D**) (Figure 7) shows that both structures crystallize in the triclinic system, P1 space group with following cell parameters: 4.8000(2) Å, 7.6568(3) Å, 9.6384(4) Å, 95.910(3)°, 96.094(4)°, and 106.833(4)° for **3L** and 4.8055 (2) Å, 7.6526 (3) Å, 9.6387 (4) Å, 95.908 (3)°, 96.052 (4)°, and 106.862 (4)° for **3D**. The difference between the lengths of the cell edges (a, b, and c) of **3L** and **3D** are in the range 0.0003(6)–0.0055(3) Å. This is a multitude of esd values for such differences, however, could be still within the sample standard deviation for multiple measurements. For comparison, the corresponding differences for the rotamers of xylitol (**1** and **2**) are in the range 0.0043(2)–0.0085(3) Å. The asymmetric units of both structures, **3L** and **3D,** contain two molecules of L-arabitol and D-arabitol, respectively. Molecules in the asymmetric unit for both structures are rotamers. Although the carbon chain is not fully superimposable, the most significant rotations occur within the hydroxyl groups (Figure 8).

### 2.4. Theoretical Calculations

**Figure 9:**
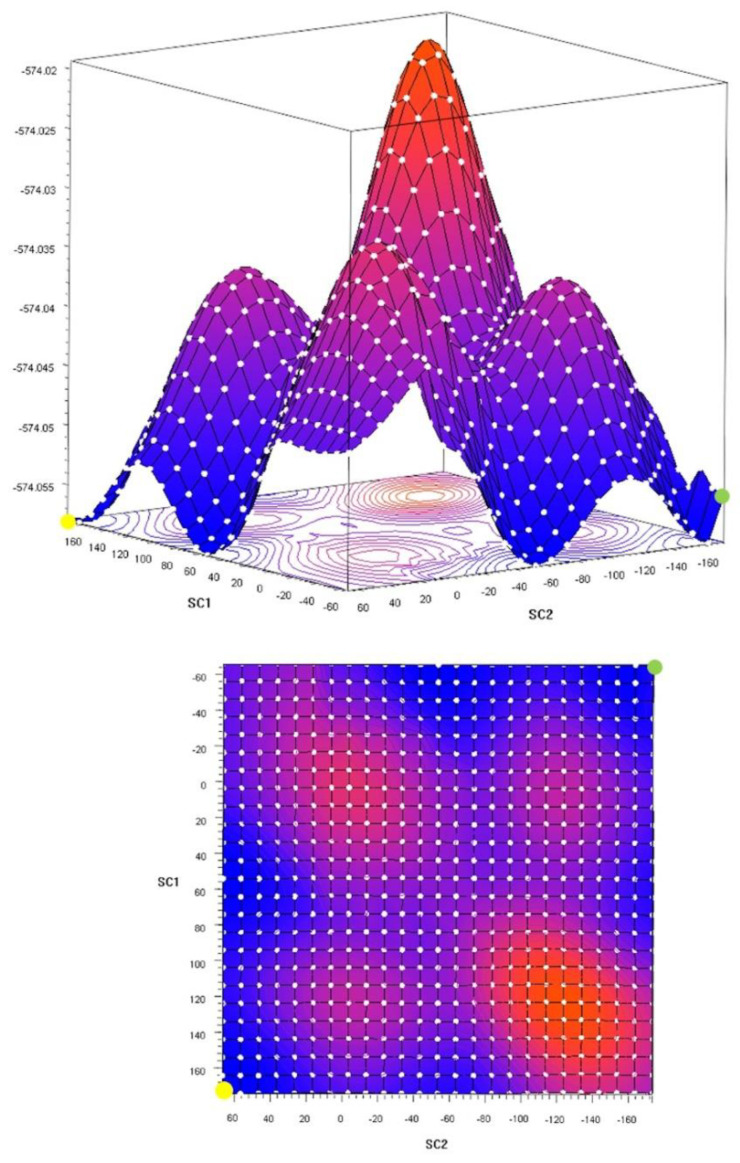
Gaussian Scan Grids (2D and 3D). Energies are equal to −574.058 Hartree and −564.054 Hartree for the rotamers **1** and **2**, respectively. The difference between them was equal to 10.5 kJ/mol. Rotamers **1** (rotation around the C2–C3 bond) and **2** (rotation around the C3–C4 bond) were marked by yellow and green points, respectively.

**Figure 10:**
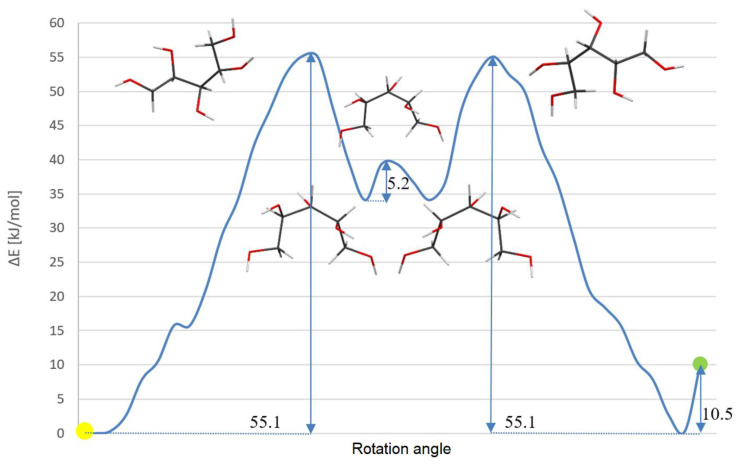
Scheme of energetic rotation barrier obtained from Gaussian09 using a scan method. Differences between energetic conformations are shown in kJ/mol. Rotamers **1** and **2** were marked by yellow and green points, respectively.

Theoretical calculations for both rotamers of xylitol were performed to establish the energetic barrier required to transform one rotamer into the other one through possible rotations of molecular fragments in the gas phase. Geometry optimization was performed to investigate preferences for any particular rotamer. Calculations performed for both rotamers of xylitol gave similar energy values differences, which was equal to ca. 10^−3^ kJ/mol, supporting the NMR spectroscopic results obtained in the solution. To further investigate the dependence of the energies on conformations, torsion angle scans were performed (Figure 9 and Figure 10). Interestingly, the scan calculations and the scheme of rotation barriers were almost symmetric. The results showed that the differences between the highest energy conformation and the energy of rotamers **1** and **2** were equal to 42.0 kJ/mol and 31.5 kJ/mol, respectively. Furthermore, according to this calculation, the difference between the rotamers **1** and **2** was equal to 10.5 kJ/mol. However, this difference could be justified by exclusion of other torsions than the two defining the scan, which increases the probability of the existence of a local minimum that would not be observed in the case of the full conformational space.

**Figure 11:**
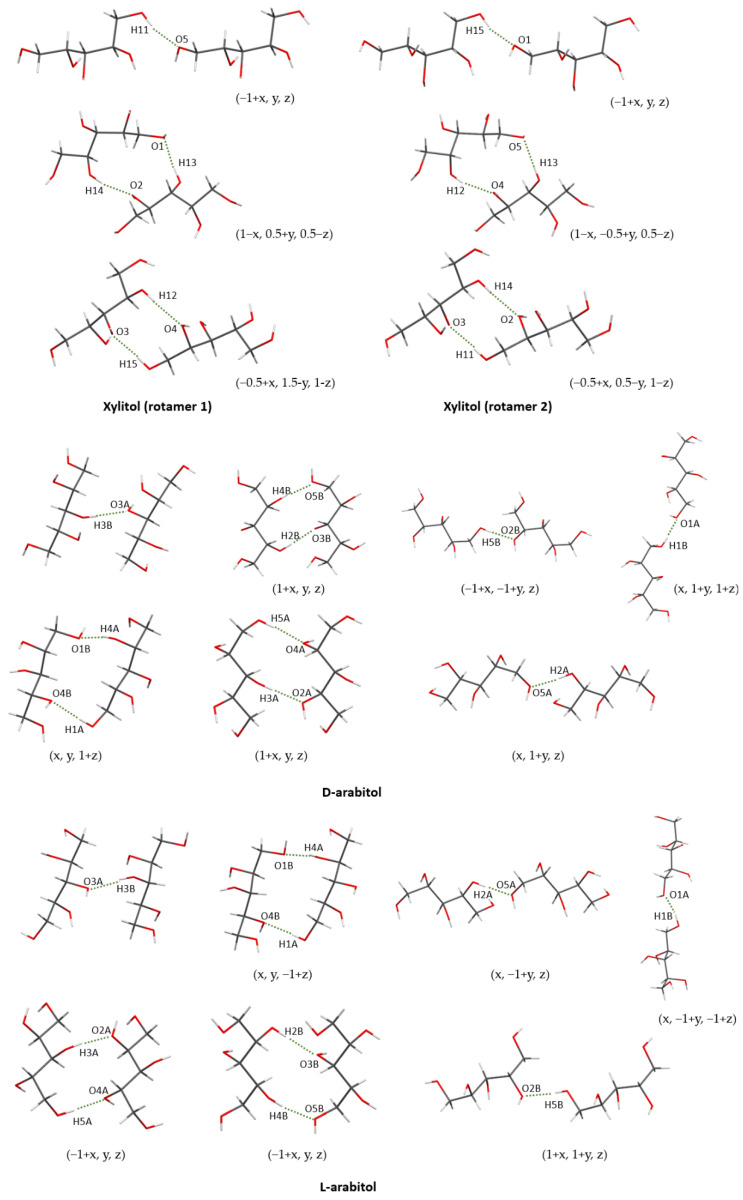
Dimers of rotamers **1** and **2** of xylitol as well as L- and D-arabitol. For clarity, only interacting atoms are assigned. Symmetry operations are given in parenthesis. Dimers O3A…H3B are formed within asymmetric unit.

The energy of dimers retrieved from the crystal structure of xylitol was calculated. Notably, dimers in both structures are not equivalent (Figure 11 and Figure S5). Although the types of hydrogen bonds and hydrogen bond motifs are the same, the donors and acceptors are opposite in both crystal structures, e.g., hydrogen bonds O4–H4…O2–H2 and O2–H2…O4–H4 were found in the crystal structures of the rotamers **1** and **2**, respectively. Nonetheless, the differences between the energy of corresponding dimers (e.g., O4–H4…O2–H2 and O2–H2…O4–H4) were not higher than 0.09 kJ/mol (Table 1). The calculations showed that both structures were almost energetically equivalent. Hence, the rotation of xylitol molecule fragments results in the formation of crystals with close stability with cohesive energies for crystal structures of rotamers **1** and **2** equal to: −452.46 kJ/mol and −452.56 kJ/mol, respectively. These results were in accordance with the results of geometrical analysis.

These calculations were repeated for D- and L-arabitol dimers (Figure 11 and Figure S5). Contrary to the xylitol, the hydrogen bonds are the same, including donors and acceptors. The energy differences between **3D** and **3L** dimers are not higher than 0.19 kJ/mol (Table 2). However, differences between donors and acceptors occur for corresponding hydrogen bonds of two molecules in the asymmetric unit, e.g., dimers with hydrogen bonds O2A–H2A…O5A–H5A and H2B–O2B…H5B–O5B were found. Differences between these corresponding dimers for both structures 3D and 3L are equal to 2.25 kJ/mol and 2.33 kJ/mol, respectively. The second pair of corresponding dimers are dimers with following hydrogens bonds: O2B–H2B…O3B–H3B/O4B–H4B…O5B–H5B and O3A–H3A…O2A–H2A/O5A–H5A…O4A–H4A. For these dimers, differences are equal to 3.51kJ/mol and 3.67 kJ/mol, respectively. The differences between structures of **3D** and **3L** were confirmed by calculation of cohesive energies and equal to 314.90 kJ/mol for **3D** and 318.54 kJ/mol for **3L**. The lattice energy difference is slightly higher for D- and L-arabtiol than rotamers of xylitol; however, the differences in theoretical calculation of **3D** and **3L** in comparison to structures **1** and **2** are a result of rotations of hydroxyl groups in **3D** and **3L** structures (Figure 8). This issue is not observed for the rotamers of xylitol as the rotations are present only in the carbon chain. Inverting the structure of one of the xylitol rotamer (with Flack parameter equal to ca. 1) and overlaying it with the second rotamer (with correct Flack parameter, equal to ca. 0) would result in the same orientation of hydroxyl groups.

### 2.5. Absolute Configuration of Xylitol

Because of a lack of anomalous-dispersion effects in the neutron measurements, the absolute configuration could be established using different methods. Only one out of the all-deposited X-ray data of xylitol (**D**) had the absolute configuration already established. The absolute configuration of others deposits was either unknown (**A**, **E**) or no information was available (**C**). However, the absolute configuration of these deposited structures and neutron data was arbitrarily established as rotamer **2**. There is no experimental evidence that **A**–**C** are really rotamer **2**. Due to the inversion relation between rotamer **1** and rotamer **2**, we inverted the structure of **1** to obtain structure **3** (Table S3) with the Flack parameter equal to 0.99(4). Comparison of the Newman projections of **3** and **D** revealed differences in the range from 0.00(1)° to 2.26(2)° (Table S4). Moreover, the results of comparison of bond lengths between **3** and **D** are similar to those resulting from comparison **1** and **D**, i.e., the range 0.000(2)–0.002(2) Å for the C-C bonds and 0.000(2)–0.007(2) Å for the C-O bonds (Table S5). These results showed that the absolute configuration of the neutron data of xylitol is unknown. The aforementioned differences between rotamer **1** (structure **1**) and rotamer **2** (structures **3** and **D**) are vague, and the neutron data could be treated as a reference for both rotamers. This is important due to fact that xylitol is one of the main compounds used in methodological studies [1,4,28]. However, we also assume that neutron data represent rotamer **2** as that crystallization was performed by the authors of structure **D**.

### 2.6. Numbering System in Deposited Compounds

The numbering schemes of carbon atoms found in structures of galactitol (CSD refcode: GALACT), ribitol (CSD refcodes: RIBITOL01 and RIBTOL02) and perseitol (CSD refcode: KAYNAZ) were the opposite of those established by IUPAC. Moreover, in some deposited structures, e.g., ALITOL01, the numbering system was not in accordance with IUPAC naming rules. Furthermore, plenty of deposited structures with more than one moiety in the asymmetric unit, e.g., VOZMAY, had the correct numbering scheme only for one moiety. We suggest that to avoid misunderstandings, the correct numbering system for all compounds in the asymmetric unit should be applied. This would allow for an easy comparison of conformations.

## 3. Materials and Methods

### 3.1. Preparation of Crystals

Xylitol (**1**) was acquired from Sigma Aldrich and crystallized from ethanol by slow cooling (0.5 °C/h) from 40 °C to 20 °C. Xylitol (**2**) was provided by Anders Ø. Madsen. Crystals of **2** were obtained from 95% ethanol using slow evaporation method [7].

We performed several experiments to obtain xylitol (**2**) including:

- Recrystallization of xylitol (**2**);
- Co-crystallization of xylitol (**1**) and xylitol (**2**);
- Co-crystallization of xylitol (**1**) with other sugars (i.e., D-arabitol, L-arabitol, and ribitol).

All above approaches were performed with the following solvents: water, ethanol, water-ethanol (1:1), and isopropanol. Compounds were dissolved in 70 °C and crystallized from 65 °C to 5 °C by slow cooling (0.5 °C/h). For each experiment, we obtained crystals of xylitol (**1**). Additionally, we acquired D-arabitol and L-arabitol from Sigma Aldrich (Darmstadt, Germany) and crystallized from isopropanol by slow evaporation.

### 3.2. Single Crystal X-ray Studies

The CuK_α_ X-ray diffraction data for xylitol (**1** and **2**), L-arabitol (**3L**), and D-arabitol (**3D**) were collected using Supernova Dual Agilent 4-circle X-ray diffractometer equipped with an Atlas detector. The instrument was run with the Xcalibur software and equipped with an CuK_α_ X-ray tube (λ = 1.54184 Å, 50.0 kV, 0.8 mA). The measurements were carried out at 122 K, 123 K, 100 K, and 100 K, respectively, using an Oxford Cryostream cooling device. Pieces of analyzed single crystals were positioned at 73 mm, 72 mm, respectively, from the CCD camera. The 5704, 3718, 5859 and 1450 frames, respectively, measured at 1° intervals with a counting time of 1s, were collected.

The refinement was based on *F*^2^ for all reflections except those with negative intensities. Weighted R factors (*w*R) and all goodness-of-fit (GooF) values were based on *F*^2^. Conventional R factors were based on the amplitudes, with *F* set to zero for negative *F*^2^. The *F_o_*^2^ > 2σ(*F_o_*^2^) criterion was applied only for R factor calculations and was irrelevant to the choice of reflections for the refinement. The R factors based on *F*^2^ were about twice as large as those based on *F* for all structures. Scattering factors were taken from the International Crystallographic Tables Vol. C [29]. Crystal data, data collection and refinement details for all compounds are presented in Table 3.

### 3.3. CSD Search

Search of the Cambridge Structural Databases version 5.39 (Cambridge, United Kingdom) [33] resulted in five structures of xylitol (CSD refcodes: XYLTOL [17] (**A**), XYLTOL01 [7], determined by neutron diffraction (**B**), XYLTOL02 [6] (**C**), XYLTOL03 [5] (**D**), and XYLTOL04 [1] (**E**)). The Flack parameter was only reported for structure **D** and was equal to 0.0(2). Therefore, molecule **D** (measured at 122 K) was used as a reference in this work. As **E** was the structure obtained from **D** after HAR (Hirshfeld Atom Refinement) [34], the absolute configuration of both of these structures was the same. Moreover, hkl files were deposited only for **B** and **D,** and both were merged according to point groups mmm and 222, respectively. Several other crystal structures of sugar alcohols were also examined, and we will discuss the numbering system of following crystal structures (CSD refcodes): GALACT, RIBITOL01, RIBTOL02, KAYNAZ, ALITOL01, and VOZMAY.

### 3.4. Computational Studies

Calculations for molecule **1** and the reference molecule **D** were performed with the aid of Gaussian09 software (Gaussian, Inc., Wallingford, CT, USA) [35] and CRYSTAL17 program (CRYSTAL - Theoretical Chemistry Group, Torino, Italy) [36,37]. The H-X lengths of bonds were extended to the mean neutron values [38] using LSDB program [39,40]. Shortened H-X bond lengths after IAM led to unreliable results of theoretical computations. Optimization and cohesive energy calculations were performed using CRYSTAL17 [36,37] at the DFT/B3LYP level of theory and applying the DFT-D3(ATM) dispersion correction [41]. The cohesive energy values were calculated as the difference between the crystal lattice energy per molecule and the energy of the isolated molecule in the gas phase, including the Basis Set Superposition Error (BSSE). Some ghost atoms used for BSSE estimation were selected up to 4 Å from the central molecule [42]. Scans of some selected torsion angles of xylitol in the gas phase using DFT methods with the B3LYP functional and 6-311++g(2d,2p) basis set (B3LYP/6-311g(2d,2p)) were also performed. The scans were performed for two torsion angles, C1−C2−C3−C4 and C2−C3−C4−C5, of the molecule **D** with 10° steps. The scans were completed after reaching the values of torsion angles equal to those in **1**. The dimer energies were calculated using DFT methods and the M06-2X functional and cc-pVDZ basis sets because testing revealed that these parameters gave the most accurate results for the binding energies of noncovalent interactions [43]. BSSE correction was accomplished by applying the standard counterpoise correction [44,45]. The energy of dimers was calculated starting from the optimized structures.

### 3.5. Powder X-ray Diffraction Studies

A powder sample of xylitol for powder X-ray diffraction studies was prepared from recrystallized xylitol acquired from Sigma Aldrich. X-ray powder diffraction study was performed at ambient temperature using a Bruker D8 Discover diffractometer equipped with a linear position-sensitive VANTEC detector. Data was collected using CuK(α_1_) radiation, in the 2θ range from 10° up to 50° with 0.012223°, 0.5 s step. Two sets of powder diffraction patterns were calculated using Mercury Software (Cambridge, United Kingdom) [46] for the non-optimized structures. The Cif files used for first set contain original cell parameters (Rotamer **1**: *a* = 8.26640(10) Å, *b* = 8.89780(10) Å, *c* = 8.9132(2) Å; Rotamer **2**: *a* = 8.264(4) Å, *b* = 8.901(2) Å, and *c* = 8.9223(14) Å), and the cif files used for the second set contain the same average cell parameters (*a* = 8.265(2) Å, *b* = 8.900(1) Å, and *c* = 8.918(2) Å).

## 4. Conclusions

We described the crystal and molecular structure of xylitol rotamers, D-arabitol, and L-arabitol. Xylitol is an example of chiral crystal of a *meso* molecule. According to our studies, only data with anomalous dispersion may be trustworthy to establish the conformation of analyzed compounds. The unit cell dimensions may be almost identical for the not superposable compounds—as we showed in the examples of crystal structures of rotamers of xylitol and crystal structures of D-arabitol and L-arabitol.

Most rotamers, including D-arabitol and L-arabitol, only slightly differ in the carbon chain and significantly differ by the rotations of the hydroxyl group. However, xylitol rotamers behave differently. Rotation occurs around C2−C3 (rotamer **1**) or C3−C4 bond (rotamer **2**), starting from the linear conformer. This difference is also reflected in the lattice energy calculations. Due to lack of rotations of hydrogen groups, both rotamers of xylitol result in similar energies of bulk and dimer motifs. Corresponding calculations for D- and L-arabitol crystal structure result in slightly higher differences due to the change of hydrogen bond network.

## Figures and Tables

**Table 1:** Dimer energy calculations using M06-2X/cc-pVDZ for xylitol rotamers **1** and **2** by counterpoise method in Gaussian09. Symmetry operations are given in brackets.

Structure	Dimers	Gaussian Calculations (kJ/mol)
Rotamer 1 (**1**)	H12…O4_(−0.5+x, 1.5−y, 1−z)_ O3…H15_(−0.5+x, 1.5−y, 1−z)_	−48.37
	H14…O2_(1−x, 0.5+y, 0.5−z)_ O1…H13_(1−x, 0.5+y, 0.5−z)_	−50.56
	H11…O5_(−1+x, y, z)_	−13.28
Rotamer 2 (**D**)	H14…O2_(−0.5+x, 0.5−y, 1−z)_ O3…H11_(−0.5+x, 0.5−y, 1−z)_	−48.47
	H12…O4_(1−x, −0.5+y, 0.5−z)_ O5…H13_(1−x, −0.5+y, 0.5−z)_	−50.56
	H15…O1_(−1+x, y, z)_	−13.26

**Table 2:** Dimer energy calculations using M06-2X/cc-pVDZ for D-arabitol and L-arabitol by counterpoise method in Gaussian09. Symmetry operations are given in parenthesis. Dimers O3A…H3B are formed within asymmetric unit.

Structure	Dimers	Gaussian Calculations [kJ/mol]
D-arabitol (**3D**)	O1A…H1B_(x, 1+y, 1+z)_	−15.78
	H1A…O4B_(x, y, 1+z)_, H4A…O1B_(x, y, 1+z)_	−61.74
	O3A…H3B	−31.56
	H3A…O2A_(1+x, y, z)_, H5A…O4A_(1+x, y, z)_	−48.45
	H2A…O5A_(x, 1+y, z)_	−18.23
	O2B…H5B_(−1+x, −1+y, z)_	−20.48
	O3B…H2B_(1+x, y, z)_, O5B…H4B_(1+x, y, z)_	−51.96
L-arabitol (**3L**)	O1A…H1B_(x, −1+y, −1+z)_	−15.90
	H1A…O4B_(x, y, −1+z)_, H4A…O1B_(x, y, −1+z)_	−61.78
	O3A…H3B	−31.46
	H3A…O2A_(−1+x, y, z)_, H5A…O4A_(−1+x, y, z)_	−48.36
	H2A…O5A_(x, −1+y, z)_	−18.34
	O2B…H5B_(1+x, 1+y, z)_	−20.67
	H2B…O3B_(−1+x, y, z)_, H4B…O5B_(−1+x, y, z)_	−52.03

**Table 3:** Crystal data and experimental details.

Crystal Data	1	2	3L	3D
Chemical formula	C_5_H_12_O_5_			
*M*_r_	152.15			
Crystal system, space group	Orthorhombic, *P*2_1_2_1_2_1_	Triclinic, P1		
Temperature (K)	122	123	100	100
*a*, *b*, *c* (Å)	8.2664(1), 8.8978(1), 8.9132(2)	8.2707(2), 8.9022(2), 8.9217(2)	4.8000(2), 7.6568(3), 9.6384(4), 95.910(3), 96.094(4), 106.833(4)	4.8055(2), 7.6526(3), 9.6387(4), 95.908(3), 96.052(4), 106.862(4)
*V* (Å^3^)	655.59(2)	656.88(3)	333.80(2)	333.99(3)
*Z*	4	2	2	
Radiation type	Cu K_α_			
µ (mm^−1^)	1.21	1.20	1.19	1.19
Crystal size (mm)	0.23 × 0.17 × 0.09	0.18 × 0.22 × 0.35	0.28 × 0.09 × 0.05	0.17 × 0.15 × 0.12
**Data Collection**				
Diffractometer	SuperNova, Dual, CuKα, and Atlas detector			
*T*_min_, *T*_max_	0.837, 1.000	0.383, 1.000	0.825, 1.000	0.901, 1.000
No. of measured, independent, and observed [*I* > 2σ(*I*)] reflections	23,329, 1325, and 1316	14,853, 1383, and 1370	11,435, 2336, and 2230	8367, 2449, and 2385
*R*_int_	0.026	0.046	0.027	0.027
(sin θ/λ)_max_ (Å^−1^)	0.624	0.631	0.625	0.624
**Refinement**				
*R*[*F*^2^ > 2σ(*F*^2^)], *wR*(*F*^2^), *S*	0.022, 0.057, and 1.07	0.028, 0.077, and 1.09	0.032, 0.084, and 1.05	0.028, 0.074, and 1.04
No. of reflections	1325	1383	2336	2449
No. of parameters	102	99	277	191
H-atom treatment	H atoms treated by a mixture of independent and constrained refinement			
Δρ_max_, Δρ_min_ (e Å^−3^)	0.27, −0.15	0.32, −0.21	0.33, −0.22	0.30, −0.18
Absolute structure	Flack × determined using 525 quotients [(I+) − (I−)]/[(I+) + (I−)] [30]	Flack × determined using 550 quotients [(I+) − (I−)]/[(I+) + (I−)] [30]	Flack × determined using 995 quotients [(I+) − (I−)]/[(I+) + (I−)] [30]	Flack × determined using 1055 quotients [(I+) − (I−)]/[(I+) + (I−)] [30]
Absolute structure parameter	0.01(4)	0.03(7)	−0.02(13)	0.07(10)

Computer programs: CrysAlis PRO, Agilent Technologies, Version 1.171.37.34 (release 22 May 2014 CrysAlis171 .NET) (compiled 22 May 2014, 16:03:01), ShelXT [31], SHELXL [31], and Olex2 [32].

## Data Availability

Crystallographic data **1** (CCDC reference: 2076896), **2** (CCDC reference: 2076897), D-arabitol (CCDC reference: 2076894), and L-arabitol (CCDC reference: 2076895) are available.

## Supplementary Materials

The following supporting information can be downloaded at: https://www.mdpi.com/article/10.3390/ijms23073875/s1.

## References

[1] Woińska M., Grabowsky S., Dominiak P.M., Woźniak K., Jayatilaka D. (2016). Hydrogen Atoms Can Be Located Accurately and Precisely by X-Ray Crystallography. Sci. Adv..

[2] Wanat M., Malinska M., Gutmann M.J., Cooper R.I., Wozniak K. (2021). HAR, TAAM and BODD Refinements of Model Crystal Structures Using Cu Kα and Mo Kα X-Ray Diffraction Data. Acta Cryst. B.

[3] Wanat M., Malinska M., Hoser A.A., Woźniak K. (2021). Further Validation of Quantum Crystallography Approaches. Molecules.

[4] Hoser A.A., Madsen A.Ø. (2017). Dynamic Quantum Crystallography: Lattice-Dynamical Models Refined against Diffraction Data. II. Applications to l-Alanine, Naphthalene and Xylitol. Acta Cryst. A.

[5] Madsen A.Ø., Sørensen H.O., Flensburg C., Stewart R.F., Larsen S. (2004). Modeling of the Nuclear Parameters for H Atoms in X-Ray Charge-Density Studies. Acta Cryst. A.

[6] Madsen A.Ø., Mattson R., Larsen S. (2011). Understanding Thermodynamic Properties at the Molecular Level: Multiple Temperature Charge Density Study of Ribitol and Xylitol. J. Phys. Chem. A.

[7] Madsen A.Ø., Mason S., Larsen S. (2003). A Neutron Diffraction Study of Xylitol: Derivation of Mean Square Internal Vibrations for H Atoms from a Rigid-Body Description. Acta Cryst. B.

[8] Guex W., Klaeui H., Pauling H., Voirol F. (1981). Reusable Heat Devices Containing Xylitol as Heat Storage Material. U.S. Patent.

[9] Palomo Del Barrio E., Cadoret R., Daranlot J., Achchaq F. (2016). New Sugar Alcohols Mixtures for Long-Term Thermal Energy Storage Applications at Temperatures between 70 °C and 100 °C. Solar Energy Mater. Solar Cells.

[10] Paleta O., Dlouhá I., Kaplánek R., Kefurt K., Kodíček M. (2002). Novel Amphiphilic Fluoroalkylated Derivatives of Xylitol, d-Glucose and d-Galactose for Medical Applications: Hemocompatibility and Co-Emulsifying Properties. Carbohydr. Res..

[11] Zarif L., Greiner J., Pace S., Riess J.G. (1990). Synthesis of Perfluoroalkylated Xylitol Ethers and Esters: New Surfactants for Biomedical Uses. J. Med. Chem..

[12] Fronczek F.R., Kamel H.N., Slattery M. (2003). Three Polymorphs (α, β, and δ) of d-Mannitol at 100 K. Acta Cryst. C.

[13] Schouten A., Kanters J.A., Kroon J., Comini S., Looten P., Mathlouthi M. (1998). Conformational Polymorphism of D-Sorbitol (d-Glucitol): The Crystal and Molecular Structures of d-Glucitol 2/3-Hydrate and Epsilond-Glucitol. Carbohydr. Res..

[14] Park Y.J., Jeffrey G.A., Hamilton W.C. (1971). Determination of the Crystal Structure of the A Form of D-Glucitol by Neutron and X-ray Diffraction. Acta Cryst. B.

[15] Dierks T.M., Korter T.M. (2017). Comparison of Intermolecular Forces in Anhydrous Sorbitol and Solvent Cocrystals. J. Phys. Chem. A.

[16] Rukiah M., Lefebvre J., Hernandez O., van Beek W., Serpelloni M. (2004). Ab Initio Structure Determination of the Γ Form of D-Sorbitol (d-Glucitol) by Powder Synchrotron X-Ray Diffraction. J. Appl. Cryst..

[17] Kim H.S., Jeffrey G.A. (1969). The Crystal Structure of Xylitol. Acta Crystallogr. Sect. B Struct. Crystallogr. Cryst. Chem..

[18] Wolfrom M.L., Kohn E.J. (1942). Crystalline Xylitol. J. Am. Chem. Soc..

[19] Franks F., Kay R.L., Dadok J. (1988). A Nuclear Magnetic Resonance Study of Isomeric Pentitols in Aqueous and Non-Aqueous Solutions. J. Chem. Soc. Faraday Trans. Phys. Chem. Condens. Phases.

[20] Roberts J.K., Jardetzky O. (1981). Monitoring of Cellular Metabolism by NMR. Biochim. Biophys. Acta (BBA)-Rev. Bioenerg..

[21] Fenn T.D., Ringe D., Petsko G.A. (2004). Xylose Isomerase in Substrate and Inhibitor Michaelis States: Atomic Resolution Studies of a Metal-Mediated Hydride Shift. Biochemistry.

[22] Botzki A., Rigden D.J., Braun S., Nukui M., Salmen S., Hoechstetter J., Bernhardt G., Dove S., Jedrzejas M.J., Buschauer A. (2004). L-Ascorbic Acid 6-Hexadecanoate, a Potent Hyaluronidase Inhibitor: X-ray structure and molecular modeling of enzyme-inhibitor complexes. J. Biol. Chem..

[23] Safari F., Katrusiak A. (2021). Structure–Property Relationships of Molecular Shape and Orientation with Com pression and Expansion of Xylitol. Acta Cryst. B.

[24] Woinska M., Wanat M., Taciak P., Pawinski T., Minor W., Wozniak K. (2019). Energetics of Interactions in the Solid State of 2-Hydroxy-8-X-Quinoline Derivatives (X = Cl, Br, I, S-Ph): Comparison of Hirshfeld Atom, X-Ray Wavefunction and Multipole Refinements. IUCrJ.

[25] Derollez P., Guinet Y., Affouard F., Danède F., Carpentier L., Hédoux A. (2012). Structure Determination of L-Arabinitol by Powder X-Ray Diffraction. Acta Cryst. B.

[26] Carpentier L., Filali Rharrassi K., Derollez P., Guinet Y. (2013). Crystallization and Polymorphism of L-Arabitol. Thermochim. Acta.

[27] Kopf J., Morf M., Zimmer B., Köll P. (1991). Kristall- und molekülstruktur von d-arabinitol. Carbohydr. Res..

[28] Hoser A.A., Madsen A.Ø. (2016). Dynamic Quantum Crystallography: Lattice-Dynamical Models Refined against Diffraction Data. I. Theory. Acta Cryst. A.

[29] Prince E., Spiegelman C.H. (1992). International Tables for Crystallography.

[30] Parsons S., Flack H.D., Wagner T. (2013). Use of Intensity Quotients and Differences in Absolute Structure Refinement. Acta Cryst. B.

[31] Sheldrick G.M. (2015). Crystal Structure Refinement with SHELXL. Acta Cryst. C.

[32] Dolomanov O.V., Bourhis L.J., Gildea R.J., Howard J.A., Puschmann H. (2009). OLEX2: A Complete Structure Solution, Refinement and Analysis Program. J. Appl. Crystallogr..

[33] Groom C.R., Bruno I.J., Lightfoot M.P., Ward S.C. (2016). The Cambridge Structural Database. Acta Crystallogr. Sect. B Struct. Sci. Cryst. Eng. Mater..

[34] Capelli S.C., Bürgi H.-B., Dittrich B., Grabowsky S., Jayatilaka D. (2014). Hirshfeld Atom Refinement. IUCrJ.

[35] Frisch M.J., Trucks G.W., Schlegel H.B., Scuseria G.E., Robb M.A., Cheeseman J.R., Scalmani G., Barone V., Mennucci B., Petersson G.A. (2009). GAUSSIAN09.

[36] Dovesi R., Saunders V.R., Roetti C., Orlando R., Zicovich-Wilson C.M., Pascale F., Civalleri B., Doll K., Harrison N.M., Bush I.J. (2017). CRYSTAL17. CRYSTAL17 User’s Manual Torino.

[37] Dovesi R., Erba A., Orlando R., Zicovich-Wilson C.M., Civalleri B., Maschio L., Rérat M., Casassa S., Baima J., Salustro S. (2018). Quantum-Mechanical Condensed Matter Simulations with CRYSTAL. Wiley Interdiscip. Rev. Comput. Mol. Sci..

[38] Allen F.H., Bruno I.J. (2010). Bond Lengths in Organic and Metal-Organic Compounds Revisited: X—H Bond Lengths from Neutron Diffraction Data. Acta Cryst. B.

[39] Jarzembska K.N., Dominiak P.M. (2012). New version of the theoretical databank of transferable aspherical pseudoatoms, UBDB2011—Towards nucleic acid modelling. Acta Cryst. A.

[40] Volkov A., Li X., Koritsanszky T., Coppens P. (2004). Ab initio quality electrostatic atomic and molecular properties including intermolecular energies from a transferable theoretical pseudoatom databank. J. Phys. Chem. A.

[41] Grimme S., Antony J., Ehrlich S., Krieg H. (2010). A Consistent and Accurate Ab Initio Parametrization of Density Functional Dispersion Correction (DFT-D) for the 94 Elements H-Pu. J. Chem. Phys..

[42] Civalleri B., Zicovich-Wilson C.M., Valenzano L., Ugliengo P. (2008). B3LYP Augmented with an Empirical Dispersion Term (B3LYP-D*) as Applied to Molecular Crystals. CrystEngComm.

[43] DiLabio G.A., Otero-de-la-Roza A. (2016). Noncovalent Interactions in Density Functional Theory. Reviews in Computational Chemistry.

[44] Boys S.F., Bernardi F. (1970). The Calculation of Small Molecular Interactions by the Differences of Separate Total Energies. Some Procedures with Reduced Errors. Mol. Phys..

[45] Simon S., Duran M., Dannenberg J.J. (1996). How Does Basis Set Superposition Error Change the Potential Surfaces for Hydrogen-Bonded Dimers?. J. Chem. Phys..

[46] Macrae C.F., Edgington P.R., McCabe P., Pidcock E., Shields G.P., Taylor R., Towler M., Streek J.V.D. (2006). Mercury: Visualization and Analysis of Crystal Structures. J. Appl. Crystallogr..

