# Supplementary material for: Rotamers in Crystal Structures of Xylitol, D-Arabitol and L-Arabitol
Source: Int J Mol Sci. 2022 Mar 31;23(7):3875. doi: 10.3390/ijms23073875 (PMC8998848; doi:10.3390/ijms23073875)
Supplement: Supplementary file 1 [file ijms-23-03875-s001.zip › Table S1. Results soybean intercrop..pdf]

**Table S1.** Results soybean intercrop.

| | Inter-crop | Ino-culation | DM (t ha$^{-1}$) | SE (±) | LSD 5% | Weed DM (t ha$^{-1}$) | SE (±) | LSD 5% | Weed_ N (%) | SE (±) | LSD 5% | Buck-wheat N (%) | SE (±) | LSD 5% | Soybean N (%) | SE (±) | LSD 5% | Soybean N (kg ha$^{-1}$) | SE (±) | LSD 5% | No. of nodules per plant | SE (±) | LSD 5% | Weight of nodules per plant (mg) | SE (±) | LSD 5% | Nodules_ N (%) | SE (±) | LSD 5% | Nodules_ S*(%) | SE (±) | LSD 5% |
|---|---|---|---|---|---|---|---|---|---|---|---|---|---|---|---|---|---|---|---|---|---|---|---|---|---|---|---|---|---|---|---|---|
| Pi 16_17 | BS | | 3.82 | 0.15 B | 0.85 | 0.52 | 0.14 | n.s.* | | | | | | | 3.03 | 0.07 | n.s. | 20.97 | 6.82 | n.s. | 2.6 | 0.4 | n.s.* | 16.99 | 3.53 | n.s. | 5.45 | 0.19 | n.s. | 0.26 | 0.006 | n.s. |
| | S | | 1.35 | 0.27 A | | 1.56 | 0.19 | | | | | | | | 3.06 | 0.14 | | 41.52 | 16.90 | | 3.9 | 0.7 | | 26.26 | 5.49 | | 5.87 | 0.15 | | 0.25 | 0.005 | |
| | | u/u | 2.62 | 0.29 | | 0.77 | 0.18 | | | | | | | | 3.03 | 0.07 | | 32.65 | 17.53 | | 0.2 | 0.0 | | 1.12 | 0.36 A | | 5.49 | 0.34 | | 0.26 | 0.009 | |
| | | i/u | 2.77 | 0.39 | n.s. | 1.13 | 0.17 | n.s.* | | | | | | | 2.88 | 0.14 | n.s. | 27.89 | 14.41 | n.s. | 4.0 | 0.3 | n.s.* | 20.22 | 2.40 B | 13.82 | 5.77 | 0.09 | n.s. | 0.25 | 0.003 | n.s. |
| | | i/i | 2.37 | 0.23 | | 1.23 | 0.24 | | | | | | | | 3.22 | 0.10 | | 33.20 | 12.58 | | 5.6 | 0.4 | | 43.54 | 4.60 C | | 5.72 | 0.13 | | 0.25 | 0.005 | |
| Pi 17_18 | BS | | 1.89 | 0.16 A | 0.41 | 2.40 | 0.18 | n.s. | 2.04 | 0.08 | n.s. | 0.92 | 0.02 | n.s. | 2.82 | 0.15 | n.s. | 32.17 | 7.84 A | 14.3 | 14.6 | 1.9 | | 139.67 | 15.34 | n.s.* | 5.42 | 0.11 | n.s. | 0.23 | 0.005 | n.s. |
| | S | | 2.85 | 0.19 B | | 2.56 | 0.17 | | 2.15 | 0.14 | | - | - | | 2.9 | 0.17 | | 87.71 | 19.31 B | | 14.9 | 2.2 | | 150.94 | 16.48 | | 5.31 | 0.13 | | 0.23 | 0.007 | |
| | | u/u | 1.53 | 0.17 A | | 3.00 | 0.16 | | 1.98 | 0.17 | | 0.91 | 0.03 | | 1.85 | 0.08 A | | 24.68 | 19.55 A | | 2.8 | 0.4 A | | 55.87 | 6.46 | | 5.17 | 0.15 | | 0.26 | 0.006 | |
| | | i/u | 2.72 | 0.16 B | 0.47 | 2.12 | 0.14 | n.s. | 2.12 | 0.08 | n.s. | 0.94 | 0.02 | n.s. | 3.32 | 0.03 B | 0.48 | 72.54 | 24.96 B | 16.83 | 19.6 | 0.9 B | 6.02 | 189.9 | 7.77 | n.s.* | 5.28 | 0.09 | n.s. | 0.21 | 0.004 | n.s. |
| | | i/i | 2.86 | 0.14 B | | 2.32 | 0.17 | | 2.2 | 0.05 | | 0.9 | 0.03 | | 3.43 | 0.05 B | | 82.59 | 22.14 B | | 21.8 | 1.4 B | | 190.15 | 11.28 | | 5.65 | 0.09 | | 0.23 | 0.003 | |
| Eb 18_19 | BS | | 0.27 | 0.03 | | 0.08 | 0.01 | | 3.53 | 0.15 | | 2.49 | 0.09 | | 2.72 | 0.03 | | 0.81 | 0.14 | | 0.3 | 0.0 | | 0.56 | 0.11 | | | | | | | |
| | S | | 0.03 | 0.00 | n.s.* | 0.11 | 0.01 | n.s.* | 4.05 | 0.11 | n.s. | - | - | n.s. | 2.69 | 0.06 | n.s. | 0.71 | 0.13 | n.s. | 0.3 | 0.1 | n.s. | 0.76 | 0.05 | n.s. | | | | | | |
| | B | | 0.22 | 0.03 | | 0.07 | 0.01 | | 3.78 | 0.11 | | 2.23 | 0.04 | | - | - | | - | - | | - | - | | - | - | | | | | | | |
| | | u/u | 0.14 | 0.02 | | 0.10 | 0.01 | | 4.00 | 0.11 | | 2.29 | 0.13 | | 2.73 | 0.03 | | 0.69 | 0.08 | | 0.3 | 0.0 | | 1.27 | 0.06 | | | | | | | |
| | | u/i | 0.25 | 0.04 | n.s.* | 0.06 | 0.01 | n.s.* | 3.58 | 0.18 | n.s. | 2.37 | 0.02 | n.s. | 2.64 | 0.02 | n.s. | 0.80 | 0.13 | n.s. | 0.2 | 0.0 | n.s. | 0.13 | 0.02 | n.s. | | | | | | |
| | | i/u | 0.11 | 0.01 | | 0.12 | 0.01 | | 4.02 | 0.07 | | 2.22 | 0.03 | | 2.69 | 0.03 | | 0.96 | 0.10 | | 0.5 | 0.1 | | 0.65 | 0.06 | | | | | | | |
| | | i/i | 0.18 | 0.03 | | 0.06 | 0.00 | | 4.24 | 0.09 | | 2.35 | 0.03 | | 2.76 | 0.08 | | 0.60 | 0.19 | | 0.3 | 0.1 | | 0.58 | 0.15 | | | | | | | |
| | | IMc u | 0.00 | - | | 0.00 | - | | | | | | | | | | | | | | | | | | | | | | | | | |
| | | IMc i | 0.00 | - | | 0.00 | - | | | | | | | | | | | | | | | | | | | | | | | | | |
| Pa 16_17 | BS | | 0.89 | 0.04 B | | 0.36 | 0.03 | | | | | | | | 2.26 | 0.04 | | 5.57 | 1.22 A | | | | | | | | | | | | | |
| | S | | 0.44 | 0.03 A | 0.19 | 0.34 | 0.02 | n.s. | | | | | | | 2.25 | 0.04 | n.s. | 9.90 | 2.78 B | 2.28 | | | | | | | | | | | | |
| | B | | 1.02 | 0.05 B | | 0.29 | 0.03 | | | | | | | | - | - | | - | - | | | | | | | | | | | | | |
| | | u/u | 0.70 | 0.06 | | 0.32 | 0.02 | | | | | | | | 2.16 | 0.03 | | 7.00 | 2.92 | | | | | | | | | | | | | |
| | | i/u | 0.78 | 0.06 | n.s. | 0.35 | 0.03 | n.s. | | | | | | | 2.29 | 0.05 | n.s. | 6.85 | 2.02 | n.s. | | | | | | | | | | | | |
| | | i/i | 0.87 | 0.05 | | 0.32 | 0.02 | | | | | | | | 2.32 | 0.04 | | 9.36 | 2.96 | | | | | | | | | | | | | |
| Pa 17_18 | BS | | 1.25 | 0.09 B | | 0.12 | 0.01 A | | 2.39 | 0.12 | | 1.47 | 0.04 | | 2.42 | 0.11 | | 2.23 | 0.85 | | 5.5 | 0.7 B | | 35.01 | 3.90 B | | 5.13 | 0.07 B | | 0.21 | 0.002 | |
| | S | | 0.17 | 0.02 A | 0.41 | 0.40 | 0.06 B | 0.13 | 2.49 | 0.11 | n.s. | - | - | n.s. | 2.67 | 0.09 | n.s. | 4.30 | 2.00 | 1.21 | 3.5 | 0.5 A | 1.64 | 12.82 | 1.70 A | 7.77 | 4.81 | 0.04 A | 0.20 | 0.22 | 0.002 | n.s. |
| | B | | 2.09 | 0.14 C | | 0.11 | 0.01 A | | 2.87 | 0.08 | | 1.31 | 0.04 | | - | - | | - | - | | - | - | | - | - | | - | - | | - | - | |
| | | u/u | 1.03 | 0.15 | | 0.17 | 0.03 | | 2.31 | 0.12 | | 1.34 | 0.06 | | 1.99 | 0.10 A | | 3.08 | 2.16 | | 0.4 | 0.1 A | | 4.05 | 0.80 A | | 4.64 | 0.03 A | | 0.21 | 0.002 | |
| | | i/u | 1.31 | 0.18 | n.s. | 0.32 | 0.06 | n.s. | 2.88 | 0.07 | n.s. | 1.55 | 0.04 | n.s. | 2.83 | 0.08 B | 0.55 | 4.15 | 1.58 | n.s. | 7.0 | 0.4 B | 2.88 | 35.38 | 3.20 B | 9.75 | 5.24 | 0.05 B | 0.28 | 0.22 | 0.002 | n.s. |
| | | i/i | 1.17 | 0.17 | | 0.14 | 0.01 | | 2.55 | 0.12 | | 1.29 | 0.03 | | 2.83 | 0.04 B | | 2.56 | 0.75 | | 6.1 | 0.5 B | | 32.32 | 3.20 B | | 5.03 | 0.07 B | | 0.22 | 0.002 | |
| Pa 18_19 | BS | | 1.46 | 0.09 B | | 0.12 | 0.01 A | | 2.72 | 0.15 | | 1.45 | 0.05 | | 1.91 | 0.05 A | | 6.06 | 2.52 A | | 2.5 | 0.4 A | | 7.43 | 0.001 | | 4.72 | 0.05 | | 0.21 | 0.003 | |
| | S | | 0.68 | 0.07 A | 0.29 | 0.43 | 0.02 B | 0.07 | 3.02 | 0.06 | n.s.* | - | - | n.s. | 2.22 | 0.05 B | 0.22 | 14.57 | 6.72 B | 2.99 | 3.3 | 0.5 B | 0.76 | 7.69 | 0.001 | n.s.* | 4.78 | 0.10 | n.s. | 0.22 | 0.003 | n.s. |
| | B | | 1.56 | 0.11 B | | 0.07 | 0.01 A | | 3.00 | 0.07 | | 1.55 | 0.04 | | - | - | | - | - | | - | - | | - | - | | - | - | | - | - | |
| | | u/u | 1.17 | 0.16 | | 0.21 | 0.04 | | 2.62 | 0.15 | | 1.45 | 0.06 | | 2.1 | 0.08 | | 4.11 | 0.98 A | | 0.1 | 0.0 A | | 0.22 | 0.000 | | 4.39 | 0.07 A | | 0.21 | 0.004 | |
| | | i/u | 1.18 | 0.07 | n.s. | 0.20 | 0.03 | n.s. | 2.98 | 0.06 | n.s.* | 1.52 | 0.05 | n.s. | 2.03 | 0.05 | n.s. | 12.99 | 5.38 B | 4.96 | 3.7 | 0.3 B | 1.81 | 11.86 | 0.001 | n.s.* | 5.10 | 0.10 C | 0.08 | 0.22 | 0.003 | n.s. |
| | | i/i | 1.34 | 0.09 | | 0.21 | 0.03 | | 3.14 | 0.05 | | 1.53 | 0.03 | | 2.07 | 0.03 | | 13.85 | 6.06 B | | 4.9 | 0.3 B | | 10.6 | 0.001 | | 4.75 | 0.08 B | | 0.22 | 0.003 | |

Values followed by different letters are significantly different at LSD 5 %. In this context, each site and the type of intercrop as well as inoculation was considered separately. SE = standard error; n.s. = not significant; BS = buckwheat/soybean; S = soybean; B = buckwheat; u = uninoculated; i = inoculated; IMc = inoculation main crop; DM = dry matter; N = nitrogen; S* = sulfur

*high number of outliers, normal distribution is not given
