# Supplementary material for: Rotamers in Crystal Structures of Xylitol, D-Arabitol and L-Arabitol
Source: Int J Mol Sci. 2022 Mar 31;23(7):3875. doi: 10.3390/ijms23073875 (PMC8998848; doi:10.3390/ijms23073875)
Supplement: Supplementary file 1 [file ijms-23-03875-s001.zip › Table S3. Results Eb 18_19 soybean main crop.pdf]

Tabel S3. Results Eb 18_19 soybean main crop.

	Inter-crop	Ino-culation	Yield (t ha^{-1})	SE (±)	LSD 5%		Grain N (%)	SE (±)	LSD 5%		No. of nodules per plant	SE (±)	LSD 5%		Weight of nodules per plant (mg)	SE (±)	LSD 5%	No. of nodules per plant m.r.	SE (±)	LSD 5%		No. of nodules per plant l.r.	SE (±)	LSD 5%	Shoot_ Mg (%)	SE (±)	LSD 5%	Shoot_ Ca (%)	SE (±)	LSD 5%		Shoot_ K (%)	SE (±)	LSD 5%		Shoot_ P (%)	SE (±)	LSD 5%	SPAD	SE (±)	LSD 5%	
Eb 18_19 N=32	BS		2.28	0.10			4.48	0.14			8.1	1.80			76.09	17.76		14.1	3.43			25.8	5.57		0.38	0.007		1.39	0.023		B	1.94	0.025		AB	0.19	0.003		30.0	0.72		
	S		2.27	0.11	n.s.		4.87	0.14	n.s.		6.9	1.38	n.s.		79.58	15.14	n.s.*	14.5	2.74	n.s.		20.6	4.50	n.s.*	0.35	0.004	n.s.	1.25	0.014	0.09	A	1.85	0.021	0.12	A	0.17	0.003	n.s.	29.2	0.69	n.s.	
	B		2.17	0.10			4.75	0.17			10.2	2.17			84.07	19.20		24.8	5.36			29.4	6.63		0.37	0.006		1.30	0.023		AB	1.97	0.019		AB	0.19	0.003		28.4	0.89		
	C		2.63	0.10			4.94	0.11			7.2	1.67			67.31	16.37		14.1	3.64			26.3	6.17		0.37	0.004		1.29	0.014		A	2.02	0.024		AB	0.18	0.002		33.6	1.61		
		u	1.95	0.04	0.32	A	4.16	0.03	0.4	A	0.1	0.03	3.76	A	0.46	0.05	n.s.*	0.0	0.00	7.82	A	0.2	0.09	n.s.*	0.37	0.002	n.s.	1.31	0.007	n.s.		1.90	0.013	n.s.		0.19	0.003	n.s.	28.2	2.43	3.37	A
		i	2.72	0.04		B	5.54	0.07		B	16.1	1.53		B	153.06	16.23		33.8	3.78		B	50.8	5.71		0.36	0.005		1.30	0.019			2.00	0.035			0.18	0.002		32.4	0.27		B

Values followed by different letters are significantly different at LSD 5 %. In this context, each site and the type of intercrop as well as inoculation was considered separately.

BS = buckwheat/soybean; S = soybean; B = buckwheat; C = control; u = uninoculated; i = inoculated; SE = standard error; LSD = least significant difference;

N = nitrogen; m.r. = main root; l.r. = lateral root; Ca = calcium; P = phosphor; K = potassium; n.s. = not significant;

*high number of outliers, normal distribution is not given
