# Supplementary material for: Rotamers in Crystal Structures of Xylitol, D-Arabitol and L-Arabitol
Source: Int J Mol Sci. 2022 Mar 31;23(7):3875. doi: 10.3390/ijms23073875 (PMC8998848; doi:10.3390/ijms23073875)
Supplement: Supplementary file 1 [file Table S2. Results soybean main crop.pdf › Table S2. Results soybean main crop.pdf]

Table S2. Results soybean main crop.

	Inter-crop	Ino-culation	Yield (t ha^{-1})	SE (±)	LSD 5%	Grain N (%)	SE (±)	LSD 5%	No. of nodules per plant	SE (±)	LSD 5%	Weight of nodules per plant (mg)	SE (±)	LSD 5%	Nodules N (%)	SE (±)	LSD 5%	Nodules S* (%)	SE (±)	LSD 5%	No. of nodules per plant m.r.	SE (±)	LSD 5%	No. of nodules per plant l.r.	SE (±)	LSD 5%
Pi 16_17	BS		0.58	0.05 B	0.15	5.88	0.14 A		13.0	2.8	n.s.	143.54	13.75	n.s.												
	S		0.42	0.04 A		6.22	0.19 B	0.30	13.8	2.8		132.63	17.95													
		u/u	0.50	0.02 A		5.18	0.06 A		2.6	0.5 A		77.78	15.53 A													
		i/u	0.64	0.05 B	0.13	6.29	0.08 B		7.8	0.5 A	8.42	126.09	7.62 AB	95.16												
		i/i	0.54	0.04 B		6.68	0.10 B	0.51	29.9	2.1 B		210.38	8.93 B													
Pi 17_18	BS		0.25	0.01	n.s.	6.59	0.06		8.5	1.5	n.s.	29.01	5.09	n.s.	5.85	0.05	n.s.	0.38	0.03	n.s.						
	S		0.24	0.02		6.67	0.03	n.s.	11.5	1.4		43.62	5.24		5.77	0.07		0.33	0.03							
		u/u	0.20	0.02 A		6.43	0.06 A		4.4	0.7 A		21.64	4.06 A		6.00	0.06		0.36	0.04							
		i/u	0.29	0.01 B	0.05	6.77	0.03 B		7.8	1.3 B	1.39	34.38	5.76 AB	19.83	5.71	0.06	n.s.	0.31	0.02	n.s.						
		i/i	0.24	0.01 A		6.69	0.03 B	0.13	17.8	0.6 C		52.92	4.18 B		5.73	0.05		0.40	0.03							
Eb 18_19	BS		2.25	0.07		4.78	0.09		7.3	1.2		65.65	11.11								14.3	2.5		24.0	3.6	
	S		2.20	0.06	n.s.	4.86	0.09	n.s.	6.6	1.1	n.s.	65.35	10.44	n.s.*							13.4	2.0	n.s.	24.8	3.7	n.s.*
	B		2.17	0.07		4.79	0.11		7.7	1.3		70.31	11.38								17.1	3.2		21.4	4.1	
		u/u	1.89	0.04 A		4.07	0.05 A		0.1	0.0 A		0.14	0.05								0.0	0.0 A		0.2	0.1	
		u/i	2.60	0.07 B	0.21	5.57	0.03 B	0.24	16.7	1.0 C	3.89	159.69	9.64	n.s.*							35.6	2.6 C	7,42	50.3	3.4	n.s.*
		i/u	1.93	0.04 A		4.22	0.03 A		0.2	0.1 A		1.03	0.38								0.0	0.0 A		0.6	0.2	
		i/i	2.42	0.05 B		5.37	0.03 B		11.9	0.7 B		107.56	6.15								24.2	1.9 B		42.4	2.7	
		IMc u	2.51	0.03 B	0.23	4.14	0.02 A		0.1	0.0 A	2.84	1.42	0.39	n.s.*							0.0	0.0 A	6,94	0.4	0.1	n.s.*
		IMc i	1.91	0.03 A		5.57	0.05 B	0.21	14.4	1.2 B		133.18	12.27								29.9	2.9 B		46.4	4.3	
Pa 16_17	BS		2.96	0.02								41.87	5.69		6.41	0.05		0.31	0.01							
	S		3.00	0.06	n.s.							54.05	7.89	n.s.	6.49	0.04	n.s.	0.30	0.00	n.s.						
	B		2.90	0.03								53.03	6.62		6.54	0.13		0.29	0.00							
		u/u	2.87	0.02 A								35.87	6.00		6.64	0.04		0.31	0.01							
		i/u	2.89	0.02 A	0.11							66.45	7.52	n.s.	6.33	0.06	n.s.	0.31	0.01	n.s.						
		i/i	3.09	0.03 B								46.62	5.84		6.37	0.13		0.30	0.00							
Pa 17_18	BS		2.15	0.03 B																						
	S		2.31	0.03 C	0.13																					
	B		1.89	0.03 A																						
		u/u	1.97	0.03 A																						
		i/u	2.09	0.03 B	0.07																					
		i/i	2.29	0.04 C																						
Pa 18_19	BS		1.58	0.02 B																						
	S		1.69	0.03 C	0.08																					
	B		1.48	0.02 A																						
		u/u	1.47	0.02 A																						
		i/u	1.58	0.01 AB	0.12																					
		i/i	1.69	0.03 B																						

	Inter-crop	Ino-culation	SPAD	SE (±)	LSD 5%	Shoot_ Mg (%)	SE (±)	LSD 5%	Shoot_ Na (%)	SE (±)	LSD 5%	Shoot_ S* (%)	SE (±)	LSD 5%	Shoot_ Ca (%)	SE (±)	LSD 5%	Shoot_ K (%)	SE (±)	LSD 5%	Shoot_ P (%)	SE (±)	LSD 5%
Pi 16_17	BS					0.43	0.005	n.s.	0.032	0.001	n.s.*	0.091	0.003	n.s.*	1.25	0.014	n.s.	1.47	0.021	n.s.	0.24	0.004	n.s.
	S					0.44	0.008		0.031	0.001		0.090	0.005		1.24	0.016		1.41	0.020		0.23	0.005	
		u/u				0.45	0.005		0.030	0.001		0.106	0.002		1.20	0.018		1.44	0.017		0.24	0.006	
		i/u				0.42	0.004	n.s.	0.036	0.002	n.s.*	0.089	0.004	n.s.*	1.26	0.014	n.s.	1.44	0.021	n.s.	0.23	0.004	n.s.
		i/i				0.43	0.008		0.029	0.001		0.076	0.003		1.22	0.012		1.45	0.027		0.22	0.005	
Pi 17_18	BS		46.02	1.81	n.s.	0.56	0.010	n.s.	0.047	0.002	n.s.	0.113	0.003	n.s.	1.55	0.026	n.s.	1.57	0.018	n.s.	0.24	0.005	n.s.
	S		43.74	0.52		0.58	0.007		0.047	0.003		0.108	0.001		1.65	0.030		1.56	0.027		0.24	0.007	
		u/u	42.32	0.67		0.56	0.011		0.050	0.003		0.108	0.003		1.61	0.042		1.64	0.026		0.25	0.008 B	
		i/u	44.54	0.51	n.s.	0.57	0.008	n.s.	0.048	0.002	n.s.	0.113	0.003	n.s.	1.66	0.015	n.s.	1.55	0.015	n.s.	0.24	0.004 B	0.02
		i/i	47.77	2.08		0.57	0.008		0.043	0.002		0.110	0.002		1.67	0.027		1.50	0.017		0.23	0.005 A	
Eb 18_19	BS		29.96	0.55		0.37	0.004		0.045	0.001		0.089	0.001		1.35	0.014 B		2.00	0.019		0.19	0.002	
	S		29.22	0.56	n.s.	0.36	0.003	n.s.	0.046	0.001	n.s.	0.089	0.002	n.s.	1.27	0.010 A	0.05	1.91	0.021	n.s.	0.18	0.002	n.s.
	B		28.42	0.68		0.37	0.004		0.046	0.001		0.092	0.002		1.27	0.015 A		1.98	0.018		0.18	0.002	
		u/u	26.76	0.34		0.37	0.003		0.048	0.001		0.092	0.001		1.33	0.018		1.86	0.014		0.19	0.002	
		u/i	31.64	0.59	n.s.	0.36	0.005	n.s.	0.046	0.001	n.s.	0.088	0.001	n.s.	1.30	0.012	n.s.	1.99	0.019	n.s.	0.18	0.002	n.s.
		i/u	28.69	0.67		0.37	0.004		0.045	0.001		0.091	0.001		1.25	0.010		2.02	0.023		0.19	0.002	
		i/i	33.07	0.32		0.37	0.003		0.043	0.001		0.090	0.002		1.30	0.009		1.99	0.019		0.18	0.002	
		IMc u	27.73	1.83 A	2.83	0.36	0.002	n.s.	0.047	0.001	n.s.	0.090	0.001	n.s.	1.31	0.005	n.s.	1.92	0.010	n.s.	0.19	0.002	n.s.
		IMc i	32.35	0.20 B		0.37	0.004		0.044	0.001		0.091	0.001		1.28	0.015		2.00	0.026		0.18	0.001	

Values followed by different letters are significantly different at LSD 5 %. In this context, each site and the type of intercrop as well as inoculation was considered separately. SE = standard error;
n.s. = not significant; BS = buckwheat/soybean; S = soybean; B = buckwheat; u = uninoculated; i = inoculated; IMc = inoculation main crop;
m.r. = main root; l.r. = lateral root; N = nitrogen; S* = sulfur; Mg = magnesium; Na = sodium; Ca = calcium; K = potassium; P = phosphorus;
*high number of outliers, normal distribution is not given
